# Sex-related alterations of gut microbiota composition in the BTBR mouse model of autism spectrum disorder

**DOI:** 10.1038/srep45356

**Published:** 2017-03-28

**Authors:** Lorena Coretti, Claudia Cristiano, Ermanno Florio, Giovanni Scala, Adriano Lama, Simona Keller, Mariella Cuomo, Roberto Russo, Raffaela Pero, Orlando Paciello, Giuseppina Mattace Raso, Rosaria Meli, Sergio Cocozza, Antonio Calignano, Lorenzo Chiariotti, Francesca Lembo

**Affiliations:** 1 Institute of Endocrinologia ed Oncologia Sperimentale, IEOS, Consiglio Nazionale delle Ricerche CNR, Via S. Pansini, 5, 80131, Naples, Italy; 2 Department of Pharmacy, University of Naples Federico II, Via D. Montesano, 49, 80131, Naples, Italy; 3 Department of Medicina Molecolare e Biotecnologie Mediche, University of Naples Federico II, Via S. Pansini, 5, 80131, Naples, Italy; 4 Istituto Nazionale di Fisica Nucleare, Sezione di Napoli, Naples, Italy; 5 Department of Veterinary Medicine and Animal Production, University of Naples Federico II, Napoli, Italy

## Abstract

Alterations of microbiota-gut-brain axis have been invoked in the pathogenesis of autism spectrum disorders (ASD). Mouse models could represent an excellent tool to understand how gut dysbiosis and related alterations may contribute to autistic phenotype. In this study we paralleled gut microbiota (GM) profiles, behavioral characteristics, intestinal integrity and immunological features of colon tissues in BTBR T + tf/J (BTBR) inbred mice, a well established animal model of ASD. Sex differences, up to date poorly investigated in animal models, were specifically addressed. Results showed that BTBR mice of both sexes presented a marked intestinal dysbiosis, alterations of behavior, gut permeability and immunological state with respect to prosocial C57BL/6j (C57) strain. Noticeably, sex-related differences were clearly detected. We identified *Bacteroides, Parabacteroides, Sutterella, Dehalobacterium* and *Oscillospira* genera as key drivers of sex-specific gut microbiota profiles associated with selected pathological traits. Taken together, our findings indicate that alteration of GM in BTBR mice shows relevant sex-associated differences and supports the use of BTBR mouse model to dissect autism associated microbiota-gut-brain axis alteration.

The ability of the enteric microbes to directly communicate with the central nervous system (CNS), to modulate brain functions and possibly influence behavior is currently under study, and the importance of a microbiota-gut-brain axis has been established^1^. Communication between the gut microbiota (GM) and brain includes several connections such as vagus nerve, gut hormones, microbial metabolites and immune system^2^. The observation of high co-morbidity between intestinal inflammatory diseases and psychiatric symptoms such as anxiety and stress^3^,^4^ and the frequent occurrence of gastrointestinal dysfunctions in autistic patients^5^ strongly corroborated the hypothesis of an implication of GM in psychiatric conditions including autistic spectrum disorders (ASD). Pivotal studies revealed improvement in both behavioral and gastrointestinal symptoms of autistic patients upon oral short-term treatment with antibiotics and probiotics^6^,^7^, supporting a role of GM in ASD. Although evidence of a deviation of GM composition in ASD patients with respect to normally developing individuals has been reported^8^,^9^,^10^,^11^,^12^,^13^,^14^, to date no clear-cut conclusion was reached on the association of specific bacterial profiles with ASD. The use of mouse models of ASD may help to clarify the underlying mechanisms involved in communication of enteric microbes with CNS and possibly generating abnormal behaviors. The composition of GM in mice displaying features of ASD was analyzed in models of environmental risk factors such as *in utero* valproic acid (VPA) exposure and maternal immune activation (MIA)^15^,^16^. *In utero* VPA exposed mice displayed altered GM composition and fatty acid metabolism along with social behaviour abnormalities^15^. In addition, in the offspring of MIA mouse model, gastrointestinal barrier defects and microbiota alterations were observed, and probiotic treatment was shown to restore microbial composition and ameliorate behavioral alterations^16^. Given the complexity of autism pathogenesis involving environmental factors and multiple genes, several genetically-based mouse models of autism were developed including mutant and trasgenic mice^17^. One of the earliest and most studied animal model showing autistic-like phenotype is BTBR T + tf/J (BTBR) inbred mouse strain^18^,^19^,^20^,^21^,^22^. These animals are characterized by dysregulation of social communication^23^,^24^,^25^, occurrence of repetitive behaviour^21^,^26^,^27^, reduction in hippocampal neurogenesis^28^, alterations of BDNF signaling^29^ in addition to aberrant immune state^30^, all features that well parallel autistic phenotype. Based on these considerations we performed a comprehensive analysis of fully symptomatic BTBR mice. In particular in this study we paralleled GM profiles, behavioral characteristics such as social interaction, stereotyped and repetitive behavior, intestinal integrity and immunological features of colon tissues in adult male and female BTBR mice. Furthermore because sex differences in the majority of mouse models of psychiatric disorders have been frequently overlooked^31^, and because sex differences have been reported in gut microbiota composition^32^, we analyzed sex-related profiles of GM and correlated them with specific pathological traits, separately in male and female BTBR. Interestingly, we studied if specific bacterial taxa drive dysbiosis in BTBR females and males, and their relationship with the aleration of behavior, gut integrity and colon immunological state.

## Results and Discussion

### Overall structure of GM of male and female BTBR mice

We analyzed sex-related profiles of GM in BTBR mice, a known mouse model of autism to gain insights into relationship between autistic behavior and dysbiosis. Fecal microbiota of fully symptomatic, 12 months old, female and male BTBR (fBTBR and mBTBR, respectively; n = 6 mice each group) and female and male C57 control mice of same age (fC57 and mC57, respectively; n = 6 mice each group;) was analyzed by next generation sequencing (NGS) technology using the Illumina Miseq system. V3–V4 variable regions of the 16S rRNA gene were amplified and sequenced to characterize total bacterial population; 62,009.83 ± 33,665.39 high-quality sequences/sample were obtained from all 24 fecal samples, representing 3,250 operational taxonomic units (OTUs). The results shown were obtained considering a depth of 32,288 sequences/sample clustered in 2,740 OTUs; Good’s coverage > of 99.3% for all sequences in the four groups indicated good sequencing depth for reliable investigation of differences in fecal microbiota between BTBR and control mice. Among the 2,740 OTUs detected across any of the samples, 245 OTUs discriminated between fBTBR and fC57 mice, while 167 discriminated between mBTBR and mC57 mice. Discriminant OTUs were identified using two complementary analyses, LEfSe algorithm and Metastats comparison (Tables S1 and S2).

**Figure 1:**
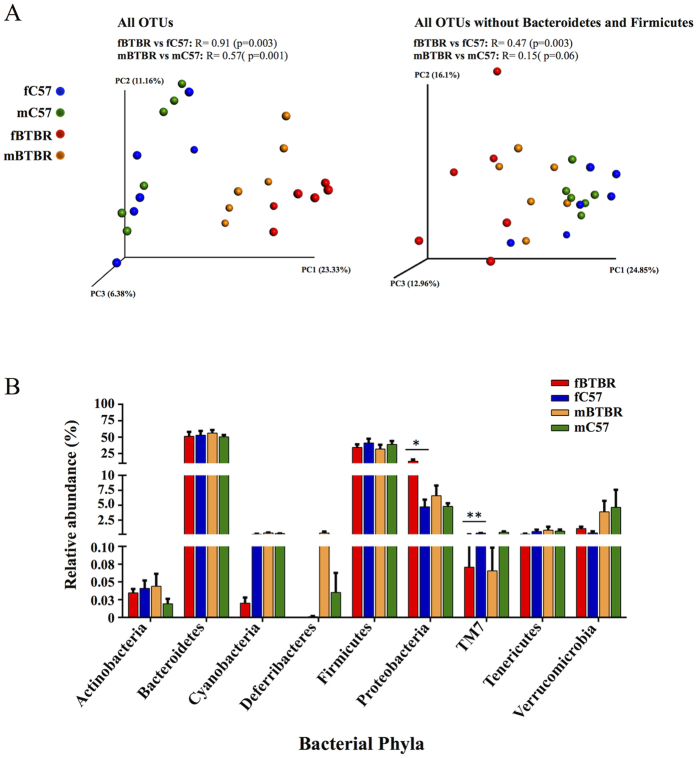
Female and male BTBR mice exhibit an altered gut microbial composition. (**A**) Unweighted UniFrac-based 3D PCoA plot constructed on all OTUs (32,288 reads per sample, left) or all OTUs without Bacteroidetes and Firmicutes reads (850 reads per sample, right) of fecal community of BTBR and C57 mice of both sexes. Analysis of similarity (ANOSIM) with 999 permutations was used to detect the statistical significant differences in microbial community composition between fBTBR and mBTBR compared to their controls (fC57 and mC57); on the top of plots are reported both R statistics and p values. (**B**) Relative abundance of all identified OTUs classified at phylum level. Mean values ± SEM are plotted (n = 6/group). Significant differences are indicated by *p < 0.05 and **p < 0.01 for comparison of fBTBR vs. fC57 and mBTBR vs. mC57. Abbreviations: fBTBR (BTBR female mice); mBTBR (BTBR male mice); fC57 (C57 female mice); mC57 (C57 male mice).

We evaluated ecological features of fecal bacterial communities in fBTBR and mBTBR compared to those of control groups. No significant differences in species richness (number of OTUs) and degree of homogeneity abundance of the species (Shannon index) were observed between groups (data not shown), while strong differences in phylogenetic assortment were detected comparing fBTBR and mBTBR with their respective controls (Fig. 1). Phylogenetic distances among samples were assessed by means of Unweighted Unifrac distance metrics, a qualitative phylogenetic measure that considers the presence/absence of a taxon. ANOSIM R statistic revealed a difference in gut bacterial assortment between BTBR of both sexes and their respective controls, with fBTBR vs fC57 displaying a higher R value compared to mBTBR vs mC57. This effect was evident in the PCoA plot, where fBTBR samples clustered to the extreme right of the plot, while mBTBR samples were positioned midway between fBTBR and control samples of both sexes (Fig. 1A, left plot).

Sequencing data revealed that 89.1% of total reads were taxonomically classified in Bacteroidetes and Firmicutes phyla, and the majority of discriminatory OTUs, both in females and males, were classified in these phyla (Tables S1 and S2). The impact of these taxa was evident when the Unweighted Unifrac analysis was repeated after negative filtering of these phyla from total sequences. After subtraction, ANOSIM analysis on remaining OTUs revealed a weaker grouping level among samples (Fig. 1A, right plot), indicating that Bacteroidetes and Firmicutes were the principal contributors to the BTBR and C57 GM differences both in female and male mice.

### GM profiling of BTBR female and male mice

Over the total of 9 bacterial phyla identified, comparison of mean abundances (by nonparametric Kruskal-Wallis test) of primary (Bacteroidetes, Firmicutes) and most of the less abundant phyla showed no significant differences between fBTBR and mBTBR compared to their respective controls (Fig. 1B). Exception was made for Proteobacteria which were found significantly more abundant in fBTBR with respect to fC57 (relative abundance 13.2% ± 2.6% and 4.7% ± 1.2%, respectively), and for TM7 phylum which was found significantly less abundant in fBTBR with respect to fC57 (relative abundance of 0.1% ± 0.04% in fBTBR and 0.3% ± 0.05% in fC57). No significant differences were found for all phyla identified in mBTBR compared to mC57 (Fig. 1B). Gram-negative Proteobacteria include lots of gut commensal species with potential pathogenic features, as the lipopolysaccharide expression. Although comparison between animal model and human disease needs to be under great scrutiny, given the known heterogeneity of ASD in both clinical and basic aspects, it is interesting to note that several families among Proteobacteria were found in the intestine of children with autism and gastrointestinal disturbances^9^. Even though the role of Proteobacteria in the pathogenesis of autism remains unclear, it is possible that alteration of commensal microbes could activate Proteobacteria as infectious agents for example through their ability to trigger immunological response.

### Key phylotypes driving GM profiles of male and female BTBR mice

In order to identify GM key phylotypes responsible for differences between fBTBR and mBTBR compared to controls we applied LEfSe algorithm. 17/30 key genera were found within Bacteroidetes and Firmicutes phyla, mainly within Bacteroidales and Clostridiales orders, even though the comparison of the relative abundances of these phyla did not show significant differences (Fig. 1B). This result confirmed that Bacteroidetes and Firmicutes taxa reassortment mainly marks the differences in the GM between BTBR and C57 mice in both sexes accordingly to ANOSIM results on Unweighted Unifrac analysis (Fig. 1A). Alteration of Clostridial and Bacteroidial OTUs was found to drive the major changes in GM composition also in maternal immune activation (MIA) model of autism^16^. Among key genera with relative abundance >0.1%, *Bacteroide*s and *Parabacteroides* (order Bacteroidales) were significantly more abundant in BTBR female and male compared to control mice (Table 1). Conversely, the genus *Dehalobacterium* (order Clostridiales) was significantly less abundant in BTBR mice of both sexes compared to controls (Table 1). Notably, the identified differences in relative abundance of *Bacteroide*s and *Parabacteroides* were more pronounced in fBTBR as indicated by fold change (Table 1). *Bacteroides* and *Parabacteroides* genera are producers of propionic acid as endproduct of their metabolism and previous studies reported that intraventricular injection of propionate in rats induced pathologic changes characteristic of ASD^33^. Moreover these microorganisms are lipopolysaccharide producers, and high levels of serum enodotoxin were detected in autistic patients^34^. Finegold *et al*.^8^ also detected high levels of *Bacteroides (Bacteroides vulgatus*) in fecal samples of severely autistic children^8^.

Among the other key genera, *Prevotella, Coprobacillus, Sutterella, Akkermansia (muciniphila*) and unclassified members of Desulfovibrionaceae and Enterobacteriaceae significantly increased, while *Oscillospira* and members of Rikenellaceae and TM7 (*AF12* and U. F16, respectively) significantly decreased in fBTBR, possibly driving a female-specific microbial signature in BTBR mice. Key genera specifically altered in mBTBR were *Lactobacillus, Ruminococcus, Desulfovibrio* and unclassified member of Helicobacteriaceae (Table 1). The alteration in the abundance of key genera that we mainly found in fBTBR was consistent, for a subset of specific taxa, with previously reported data both in human ASD patients and in a different murine model of ASD^9^,^10^,^13^,^15^. Genus *Sutterella,* within Proteobacteria, was found to be elevated both in feces of ASD children and in intestinal biopsies from children with autism and intestinal disturbances^35^,^36^. Furthermore, high levels of *Akkermansia muciniphila* were reported in BTBR fed a chow diet, while the ketogenic diet resulted in a normalization of its levels in association with improvement of behavioral symptoms^37^. Genus *Oscillospira* accounted for 13.7% in fC57 and was found significantly reduced to 5.4% in fBTBR. *Oscillospira,* recenlty identified by 16S RNA sequencing, is considered an enigmatic component of the human gut microbiota and, possibly based on butyrate production it was positively associated with human health^38^. Interestingly butyrate has recently been shown to improve repetitive behavior in BTBR mice^39^, thus it would be of interest to functionally characterize *Oscillospira,* and individual species in this genus, to examine their impact on autistic phenotype.

### Correlations between GM alterations and behavioral phenotype, gut permeability and cytokine expression in female and male BTBR mice

Along with description of BTBR GM profile we investigated the possible correlation between the levels of specific bacterial taxa and peculiar pathological traits dysregulated in ASD patients such as behavioral abnormalities, gut permeability and immune abnormalities^5^,^40^.

**Figure 2:**
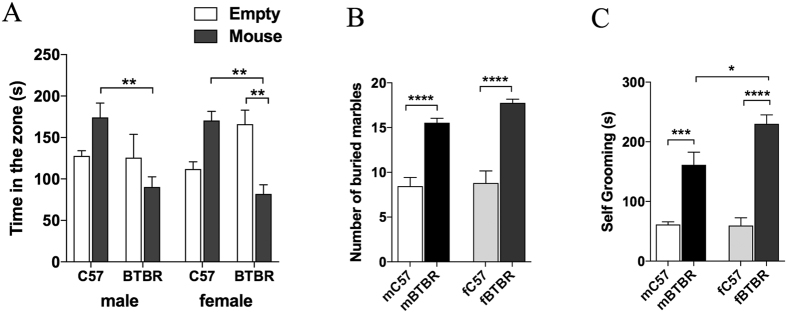
Analysis of social interaction, stereotyped and repetitive behavior in female and male BTBR mice. (**A**) Three-chamber social interaction test showing time spent in each chamber by BTBR and C57 mice of both sexes (n = 12/group; p = 0.7424 for chamber, p = 0.0077 for strain, p = 0.7761 for sex, p < 0.0001 for chamber x strain and p = 0.1671 for chamber x strain x sex, by three-way ANOVA). (**B**) Number of buried marble by BTBR and C57 mice of both sexes after a 30 min testing session (n = 12/group; p < 0.0001 for strain, p = 0.1322 for sex and p = 0.2689 for strain x sex, by two-way ANOVA). (**C**) Seconds spent in repetitive grooming measured for BTBR and C57 mice of both sexes during 10 min test session (n = 12/group; p < 0.0001 for strain, p = 0.0372 for sex, and p = 0.0269 for strain x sex, by two-way ANOVA). Significant differences are indicated by *p < 0.05, **p < 0.01, ***p < 0.001 and ****p < 0.0001 using Bonferroni post-hoc tests following three-way ANOVA with chamber, strain and sex as factors (**A**) or two-way ANOVA with strain and sex as factors (**B** and **C**). Abbreviations: fBTBR (BTBR female mice); mBTBR (BTBR male mice); fC57 (C57 female mice); mC57 (C57 male mice). Data are shown as mean values ± SEM.

Three chamber paradigm, marble burying and spontaneous self-grooming tests were used to evaluate social, stereotyped and repetitive behavior in control and BTBR mice. For all behavioral tests, both female and male BTBR mice showed deficits compared to controls, however a significant sex-related alteration was observed with fBTBR displaying higher self-grooming scores (Fig. 2A–C).

**Figure 3:**
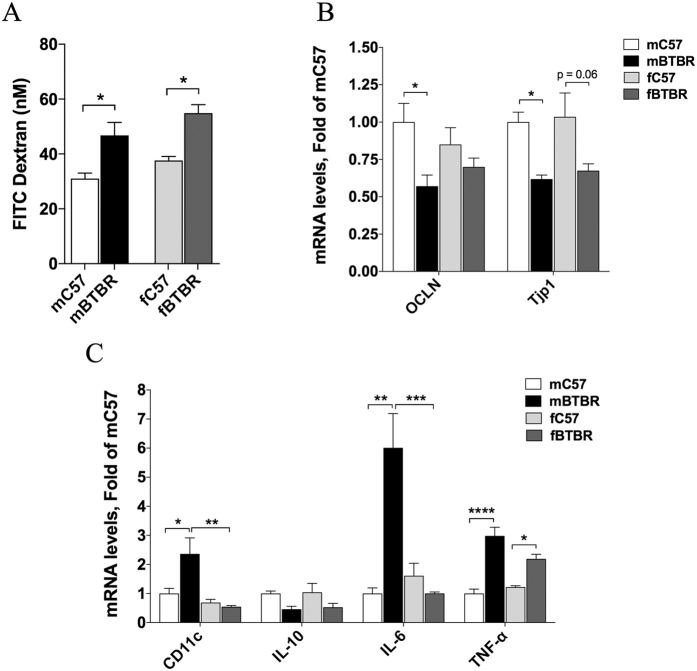
Analysis of intestinal integrity and inflammation levels in female and male BTBR mice. (**A**) Intestinal epithelial permeability to fluorescein isothiocyanate (FITC)-dextran 4 kDa of BTBR and C57 mice of both sexes. Data are represented as plasma concentration of FITC dextran (nM) (n = 5/group; p = 0.0002 for strain, p = 0.0469 for sex and p = 0.8187 for strain x sex, by two-way ANOVA). (**B**) Colon occludin (OCLN) and zonuline-1 (Tjp1), gene expression normalized to GAPDH gene in BTBR and C57 mice of both sexes. Data were normalized to mC57 control (n = 6/group; OCLN: p = 0.0067 for strain, p = 0.9105 for sex and p = 0.1623 for strain x sex; Tjp1: p = 0.0004 for strain, p = 0.6202 for sex and p = 0.9105 for strain x sex, by two-way ANOVA). (C) Colon mRNA levels of inflammatory markers (CD11c, IL-10, IL-6 and TNF-alpha) normalized to GAPDH in BTBR and C57 mice of both sexes. Data for each gene were normalized to mC57 controls (n = 6/group; CD11c: p = 0.0529 for strain, p = 0.0018 for sex and p = 0.019 for strain x sex; IL-10: p = 0.0041 for strain, p = 0.7827 for sex and p = 0.9739 for strain x sex; IL-6: p = 0.0106 for strain, p = 0.0107 for sex and p = 0.002 for strain x sex; TNF-alpha: p < 0.0001 for strain, p = 0.2196 for sex and p = 0.0354 for strain x sex, by two-way ANOVA). In bar charts all data are expressed as mean values ± SEM; significant differences are indicated by *p < 0.05, **p < 0.01, ***p < 0.001 and ****p < 0.0001; near-significant differences are also reported (Bonferroni post-hoc tests following two-way ANOVA with strain and sex as factors). Abbreviations: fBTBR (BTBR female mice); mBTBR (BTBR male mice); fC57 (C57 female mice); mC57 (C57 male mice).

**Figure 4:**
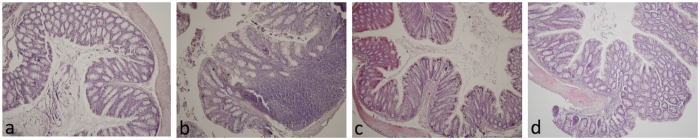
Histological evaluation of colon inflammatory cell infiltration in female and male BTBR mice. Representative hematoxylin and eosin–stained sections from colon tissues of mice. (**a**) Colon tissue from mC57 mice showing absence of inflammatory cells. (**b**) Colon tissue of mBTBR group showing leukocyte infiltration in the mucosa and submucosa. (**c**) Colon tissue of fC57 group, showing absence of inflammatory cells. (**d**) Colon tissue from fBTBR group showing moderate leukocyte infiltration in the mucosa. Original magnification 10x. Histological evaluation of inflammatory cells infiltration was scored along the entire colon length, inspecting the colon mucosa, submucosa and transmural areas considering the following parametres: leukocyte density (mC57 = 0.33 ± 0.58, mBTBR = 2 ± 1, fC57 = 0, fBTBR = 2.67 ± 0.58) and expansion of leukocyte infiltration (mC57 = 0.33 ± 0.58, mBTBR = 1.33 ± 0.58, fC57 = 0, fBTBR = 1.67 ± 0.58). The histologic scoring system is reported in Material and Methods section. Data reported as mean ± SD, n = 3/group.

Alteration of gut permeability, as evidenced by increased FITC-dextran translocation across the intestinal epithelium into blood, was observed both in female and male BTBR mice (Fig. 3A). Consistent with deficit in intestinal barrier integrity, a significant reduction of occludin and zonuline mRNA levels was detected in colon of male BTBR mice and a similar trend was observed in females (Fig. 3B). In addition, expression of a subset of cytokines (TNF alpha, IL-6 and IL-10) and CD11c integrin was determined in colon tissue of male and female BTBR and C57 mice. Increased expression of TNF alpha was observed in BTBR mice of both sexes (Fig. 3C). Significant increase of IL-6 and CD11c was observed in mBTBR compared to both sex-mached controls and fBTBR (Fig. 3C). These results indicate that, although with different marks, BTBR mice of both sexes present an increased gut permeability and altered cytokines pattern in colon tissue. In addition, histological evaluation of colon tissues showed tissue damage and evident inflammatory cells infiltration in both mBTBR and fBTBR (Fig. 4).

**Figure 5:**
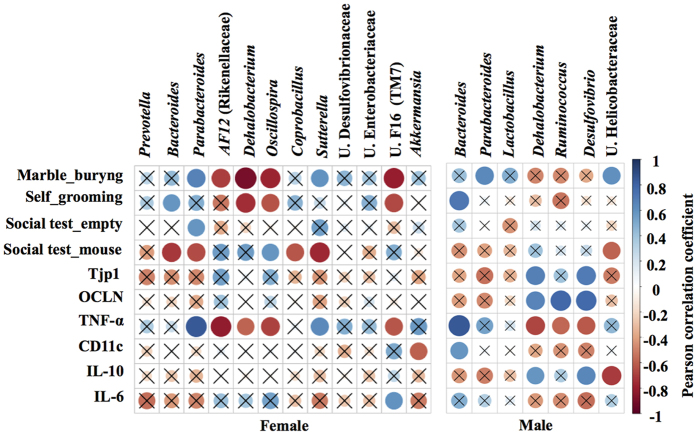
Correlogram showing the Pearson’s correlation between key genera and behavioral scores, gut integrity and immune-markers in BTBR and C57 mice of both sexes. Blue circles designate a positive correlation while red ones designate a negative correlation. With X are barred no significant results according to the significance level of 0.05.

Pearson correlation was applied to correlate the abundance of key genera, that discriminated fBTBR and mBTBR from sex-matched controls, with behavioral tests, colon mRNA expression of occludin, zonuline and immune-markers (Fig. 5). In particular, increase of *Parabacteroides* and *Sutterella,* together with decrease of *Dehalobacterium, Oscillospira* and unclassified member of TM7 were strongly associated to altered behavior and TNF-alpha expression in fBTBR. Onore and collegues (2013) already reported a relationship between the increase in inflammation levels and repetitive grooming behavior in BTBR mice^41^. Here we add the observation that remodeling of gut microbiota composition by reassortment of specific bacterial genera, may contribute to the immune dysregulation and possibly to the altered behavior of BTBR mice. Unclassified members of Helicobacteriaceae associated with abnormal behavior and low IL-10 expression in mBTBR. Finally, in mBTBR mice lower levels of *Dehalobacterium, Ruminococcus* and *Desulfovibrio* were associated with increased gut permeability (Fig. 5).

## Conclusions

Results showed that BTBR mice of both sexes presented a marked intestinal dysbiosis with respect to prosocial C57 strain. *Bacteroides, Parabacteroides, Sutterella, Dehalobacterium* and *Oscillospira* genera were identified as key drivers of sex-specific GM profiles associated with altered behavior, increased gut permeability and colon proinflammatory state. Our analysis put in evidence that definition of sex specific signatures of GM should be considered in assessing the role of GM in gut-brain axis connection. In addition, our data indicate that, despite the difficulty and limits in the use of animal models to translate human disease, BTBR mouse model is a mimic, in some relevant aspects, of autistic phenotype. Noteworthy, BTBR mice, as example of idiopathic autism, could be considered an animal model useful to investigate whether restoring GM balance may ameliorate pathological traits. A description that takes into account sex differences in GM profiles and relevant pathological traits characterizing the autistic-like phenotype, could be important for future studies aimed to translate animal findings to human diseases.

## Material and Methods

### Animals

All procedures involving animals and their care were conducted in conformity with international and national law and policies (EU Directive 2010/63/EU for animal experiments, ARRIVE guidelines and the Basel declaration including the 3R concept). The procedures reported here were approved by the Institutional Committee on the Ethics of Animal Experiments (CSV) of the University of Naples “Federico II” and by the Ministero della Salute under protocol no. 0022569-P-20/12/2010. Before killing and prior to serum and sample collection, animals, kept overnight fasted, were anesthetized by enflurane and euthanized by an intraperitoneal injection of a cocktail of ketamine/xylazine. As suggested by the animal welfare protocol, all efforts were made to minimize animal suffering and to use only the number of animals necessary to produce reliable scientific data. C57Bl/6J (C57) and BTBR T + tf/J (BTBR) inbred strains of mice were purchased from The Jackson Laboratory (Bar Harbor, ME, USA) and a colony was established and maintained. For the study, 24 fully symptomatic BTBR mice (12 months of age, 12 for gender) and 24 C57 control mice (12 months of age, 12 for gender) were housed in the same room under standard 12-h light/12-h dark cycle with free access to water and standard laboratory chow diet. Mice were from different litters and housed by gender separately 2/3 per cage.

### Marble burying test

Male and female BTBR and C57 (n = 12 each group) were individually placed in a plastic container (46 cm long by 24 cm wide by 21 cm deep) with 3 cm of clean woodchip bedding (Northeastern Products, NY) where 20 glass marbles (1.5 cm in diameter) were placed on top of clean bedding, arranged in five rows of four. Once a mouse was gently allocated into the test container, a wire lid was placed on top and mouse is allowed to freely explore the cage. After 30 minutes, each mouse was removed from the testing container and replaced to their home cage. When a threshold of 75% coverage for each marble was observed, it was considered buried and recorded. After the test, the marbles were thoroughly cleaned and new bedding was used for each mouse.

### Spontaneous self-grooming behavior

The same subjects tested in marble burying test were also examined for grooming behavior. Mice were individually allocated in an empty plastic cage (28 cm wide × 17 cm long × 12 cm high) in a separate room for a total of 20 minutes where animals were allowed to freely explore for 20 minutes. The first 10 min served as a habituation period while during the second phase of 10 min, the cumulative period that mice spent in grooming was manual scored by a trained observer. Grooming behavior included head washing, body grooming, genital/tail grooming and paw and leg licking. After the test the cage was thoroughly cleaned.

### Social approach testing

The same subjects tested in marble burying and self-grooming tests were also examined for social approach behavior. Social approach behavior was tested in a three-chambered apparatus, using methods previously described^18^,^19^. The apparatus (60 × 40 cm) has two doorways that divide it into a three chambers apparatus (20 × 40 cm each). Number of entries and time spent in each chamber were automatically detected by a videocamera coupled with a video-tracking software (Any-maze, Stoelting). The sociability test was preceded by 5-min habituation session where each mouse is restrained in the center of the middle chamber. After these phases, a novel sex, strain and age matched mouse (not used in later testing and previously habituated) is placed in one side of the chamber under an enclose cup while the other side contained an empty cup. During this sociability phase, walls between the compartments are removed and the tendency to approach a novel mouse is compared with tendency to approach a novel object. Each mouse was free to explore all three chambers for 10 minutes and both sides were alternated between the left and right chambers across subjects.

### *In vivo* intestinal permeability assay

*In vivo* intestinal permeability assay was performed for a subset of mice using fluorescein isothiocyanate-labeled dextran (FITC-dextran) method, as previously described^42^. Briefly, food and water were withdrawn for 6 h and BTBR T + tf/J and C57Bl/6 J female and male mice (n = 5, each group) were administrated by gavage with FITC labeled dextran 4000 (Sigma-Aldrich, Milan, Italy), as permeability tracer (60 mg/100 g body weight). After 24 hours blood of all animals was collected by intracardiac puncture and centrifuged (3000 rpm for 15 min at RT). Then plasma FITC-dextran concentration was determined (excitation, 485 nm; emission, 535 nm; HTS-7000 Plus-plate-reader; Perkin Elmer, Wellesley, MA, USA), using a standard curve generated by serial dilution of the tracer.

### Histological Analysis

Samples of colon from male and female mice of both strains (n = 3) were removed, washed and then fixed in paraformaldehyde (4% v/v; Carlo Erba, Italy) for 12 hours. Samples were dehydrated, embedded in paraffin and cut into 5 μm thick sections before being stained with hematoxylin-eosin (H&E; Carlo Erba, Italy). Images were obtained by a Leica DFC320 video camera (Leica, Milan, Italy) connected to a Leica DM RB microscope using the Leica Application Suite software V2.4.0. Sections were examined and scored using a 0-to-4 scale in a blinded fashion. The histologic scoring system was as follows: a) The severity of inflammatory cell infiltration was evaluated by percentage of leukocyte density in lamina propria area and estimated in a high-power field (HPF) representative of the section (0 for no signs of inflammation, 1 for minimal <10%, 2 for mild 10–25% with scattered neutrophils, 3 for moderate 26–50%, 4 for marked >51% with dense infiltrate); b) The extent of the inflammation was estimated as expansion of leukocyte infiltration (0 for none, 1 for mucosal, 2 for mucosal and submucosal and 3 for mucosal, submucosal and transmural level).

### Quantification of gene expression using RT-PCR

Colon samples were collected and immediately frozen at −80 °C until use for RNA extraction. Total RNA was extracted from colon tissues using TRIzol Reagent (Bio-Rad Laboratories), according to the manufacturer’s instructions. cDNA was synthesized using a reverse transcription kit (NucleoSpin^®^, MACHEREY-NAGEL GmbH & Co, Düren, Germany) from 4 μg total RNA. PCRs were performed with a Bio-Rad CFX96 Connect Real-time PCR System instrument and software (Bio-Rad Laboratories). The PCR conditions were 15 min at 95 °C followed by 40 cycles of two-step PCR denaturation at 94 °C for 15 s, annealing at 55 °C for 30 s and extension at 72 °C for 30 s. Each sample contained 20 ng cDNA in 2X QuantiTect SYBRGreen PCR Master Mix and primers pairs to amplify zonuline-1 (*Tjp1*), occludin (*Ocln*), TNF-α (*Tnf*), interleukin-6 (*Il6*), interleukin-10 (*Il10*), CD11c (*Itgax*) (Qiagen, Hilden, Germany), in a final volume of 50 μl. The relative amount of each studied mRNA was normalized to GAPDH as housekeeping gene, and data were analyzed according to the 2^−ΔΔCT^ method.

### Microbial DNA extraction, 16S ribosomal DNA (rDNA) library preparation and sequencing

Freshly evacuated fecal pellets were kept directly in a sterile microtube one day before the sacrifice of mice and stored at −80 °C until assayed. Bacterial genomic DNA was extracted from frozen fecal samples using the QIAamp DNA Stool Mini Kit (Qiagen) according to manufacturer’s instructions. DNA concentration was measured fluorometrically using Qubit dsDNA BR assay kit (Invitrogen) and quality was assessed by spectrophotometric measurements with NanoDrop (ThermoFisher Scientific Inc). Samples were stored at −20 °C until processed for amplification. It is well documented that various compartments of the gastro-intestinal tract harbour different bacterial populations. We chose to analyze readly accessible fecal samples for gut microbiome analyses mainly because fairly representative of the whole gastro-intestinal tract, with exception of some surface-adherent bacterial species. Sequencing samples were prepared according to the protocol 16S Metagenomic Sequencing Library Preparation for Illumina Miseq System with some modifications. The V3–V4 regions of the 16S rDNA gene were amplified starting from 200 ng of DNA template in a reaction volume of 50 μL containg 1x Fast start High Fidelity Reaction Buffer, 5 μM of each primer, 0.2 nM of dNTPs, 3 mM MgCl_2_, and 2 U FastStart High Fidelity PCR System (Roche Applied Science). PCR was performed using the following cycles conditions: an initial denaturation step at 95 °C for 2 min, followed by 30 cycles of 95 °C for 30 s, 55 °C for 45 s, 72 °C for 55 s and ended with an extension step at 72 °C for 5 minutes; products were visualized by electrophoresis on 1.2% agarose gel. After a purification step with Agencourt AMPure XP (Beckman Coulter Inc), the amplicons were indexed with 10 subsequent cycles of PCR using the Nextera XT Index Kit (Illumina). Each PCR reaction contained 10 μL of amplicons from first PCR, 5 μl index 1 primer (N7xx), 5 μl index 2 primer (S5xx), 5 μl 1x Fast start High Fidelity Reaction Buffer, 6 μL MgCl_2_ (3 mM), 1 μL dNTPs (0.2 nM), 0.4 μL FastStart High Fidelity PCR System (2U) and 17.6 μl PCR grade water. PCRs were carried out, visualized using gel electrophoresis and subsequently cleaned as described above. Library sizes were assessed using a Bioanalyzer DNA 1000 chip (Agilent technologies) and quantified with Qubit. Normalized libraries were pooled, denatured with NaOH, then diluted to 10pM and combined with 25% (v/v) denatured 10pM PhiX, according to Illumina guidelines. Sequencing run was performed on an Illumina Miseq system using v3 reangents for 2 × 281 cycles.

### Sequencing data analysis

V3-V4 16S rDNA FASTQ paired-end reads were quality filtered and assembled using PEAR^43^. Only sequences showing average PHRED score ≥30, read length between 400 and 500 bp and overlapping regions between mate-pair end of at least 40 nucleotides were retained in this step. Passing filter sequences were then processed with PRINSEQ^44^ in order to obtain FASTA and quality files for further analyses. Metagenomic analyses on the resulting data were conducted using Quantitative Insights Into Microbial Ecology (QIIME, version 1.8.0)^45^. 16S sequences were used to pick OTUs at 97% of sequences similarity from Greengenes 16S gene database (GG, may 2013 version)^46^ with a closed reference-based OTU picking method. The GG database was used to taxonomically classify the identified OTUs and to compute their distribution across different taxonomic levels. To avoid sample size biases in subsequent alpha and beta diversity analyses, a sequence rarefaction procedure was applied using a maximum depth of 32,228 sequences/sample.

To assess sampling depth coverage and species heterogeneity in each sample, alpha diversity metrics were employed on rarefied OTU table using Good’s coverage, Observed species and Shannon’s diversity index. A two-sample permutation t-test, using 999 Monte Carlo permutations to compute p-value, was performed to compare the alpha diversities between sample groups. OTUs diversity among sample communities (beta diversity) was assessed by applying unweighted Unifrac distances. Statistical significance of beta diversities was assessed on unweighted UniFrac distances matrixes using ANOSIM method^47^ with 999 permutations. Statistical differences in OTUs frequencies across sample groups at different taxonomic levels were assessed using nonparametric Kruskal-Wallis test.

Next, two analyses were applied on OTU tables generated by QIIME to identify key OTUs that discriminate female and male BTBR mice from their respective controls: Metastats comparison using the online interfaces^48^ and LDA Effect Size analysis (LEfSe)^49^. Only those OTUs reported by both methods to be significantly different between the two groups (p < 0.05 for Metastats, LDA > 2 and p < 0.05 for LEfSe) have been considered as key discriminatory OTUs. Key genera that discriminate female and male BTBR mice from their respective controls were identified applying only LEfSe.

### Other statistical methods

Marble buring, self-grooming, plasma FITC-dextran, gene expression data were analyzed by two-way ANOVA with strain and sex as factors; social approach behavior results were analyzed by three-way ANOVA with chamber, strain and sex as factors. Multiple comparisons were performed using Bonferroni’s post-hoc test. Pearson correlation test was used to assess the eventual relationship between the amount of key genera and behavioral scores, intestinal permeability and inflammation.

In this study results were considered statistically significant at p-value <0.05. Significant differences were indicated in figures by *p < 0.05, **p < 0.01, ***p < 0.001, ****p < 0.0001. ANOSIM and permutation t-test were performed using QIIME scripts, all other analyses were performed using R 3.2.0^50^. Bar plots were created by using GraphPad Prism 6.0.

## Additional Information

**How to cite this article:** Coretti, L. *et al*. Sex-related alterations of gut microbiota composition in the BTBR mouse model of autism spectrum disorder. *Sci. Rep.* **7**, 45356; doi: 10.1038/srep45356 (2017).

**Publisher's note:** Springer Nature remains neutral with regard to jurisdictional claims in published maps and institutional affiliations.

## Supplementary Material

Supplementary Table S1

Supplementary Table S2

## Figures and Tables

**Table 1:** Relative abundance of key genera discriminating female and male BTBR from their sex-matched control mice.

	fBTBR	fC57	p-value; Fold Change	mBTBR	mC57	p-value; Fold Change
*Prevotella*	4.18 ± 1.70	1.50 ± 1.20	0.037; 2.8	1.95 ± 0.50	0.72 ± 0.39	p > 0.05
*Bacteroides*	9.85 ± 3.53	0.93 ± 0.45	0.006; 10.5	6.94 ± 2.47	0.80 ± 0.16	0.004; 8.7
*Parabacteroides*	6.40 ± 1.43	0.79 ± 0.30	0.004; 8.1	4.02 ± 0.84	1.08 ± 0.36	0.025; 3.7
*AF12* (Rikenellaceae)	0.31 ± 0.07	0.76 ± 0.07	0.006; 0.4	0.57 ± 0.18	0.71 ± 0.11	p > 0.05
*Lactobacillus*	0.27 ± 0.08	0.11 ± 0.04	p > 0.05	3.48 ± 2.10	0.15 ± 0.04	0.016; 22.7
*Dehalobacterium*	0.06 ± 0.02	0.20 ± 0.04	0.025; 0.3	0.11 ± 0.04	0.27 ± 0.04	0.025; 0.4
*Oscillospira*	5.39 ± 0.68	13.71 ± 1.76	0.010; 0.4	7.16 ± 1.75	8.88 ± 0.74	p > 0.05
*Ruminococcus*	0.71 ± 0.17	1.11 ± 0.16	p > 0.05	0.68 ± 0.18	1.19 ± 0.16	0.037; 0.6
*Coprobacillus*	0.13 ± 0.07	0	0.003; 244	0.09 ± 0.03	0.01 ± 0.01	0.014; 9
*Sutterella*	4.51 ± 1.05	1.07 ± 0.48	0.016; 4.2	3.01 ± 0.87	1.71 ± 0.66	p > 0.05
U. Desulfovibrionaceae	2.78 ± 1.24	0.16 ± 0.12	0.010; 17.3	0.55 ± 0.22	0.63 ± 0.34	p > 0.05
*Desulfovibrio*	0.39 ± 0.11	1.15 ± 0.44	p > 0.05	0.11 ± 0.08	1.14 ± 0.34	0.006; 0.1
U. Helicobacteraceae	0.67 ± 0.57	0.05 ± 0.01	p > 0.05	0.13 ± 0.03	0.03 ± 0.01	0.025; 4.2
U. Enterobacteriaceae	3.38 ± 1.84	0.03 ± 0.01	0.004; 109	1.66 ± 1.11	0.13 ± 0.07	p > 0.05
U. F16 (TM7)	0.07 ± 0.04	0.29 ± 0.05	0.004; 0.2	0.07 ± 0.03	0.44 ± 0.17	p > 0.05
*Akkermansia*	1.08 ± 0.32	0.35 ± 0.27	0.016; 3.1	3.90 ± 1.85	4.62 ± 2.96	p > 0.05

Key genera were identified applying the metagenomic biomarker discovery approach of LEfSe and only genera with an LDA significant threshold >2 and relative abundance >0.1% in at least one group of mice, are shown. Fold change was expressed as ratio between the value of mean relative abundance of each genus in fBTBR and mBTBR groups and the value found in the sex-matched controls. Data are shown as average and SEM (n = 6/group).

Abbreviations: fBTBR (BTBR female mice); mBTBR (BTBR male mice); fC57 (C57 female mice); mC57 (C57 male mice).
